# POLR1D silencing suppresses lung cancer cells proliferation and migration via inhibition of PI3K-Akt pathway

**DOI:** 10.1186/s13019-024-02791-y

**Published:** 2024-06-06

**Authors:** Zhize Yuan, Yichao Wang, Yong Yang, Xiong Qin

**Affiliations:** 1 Department of Thoracic Surgery, Shanghai Pulmonary Hospital, Tongji University, No. 507, Zhengmin Road, Yangpu District, Shanghai, 200433 China; 2 Institute of Thoracic Oncology, Fudan University, Shanghai, China; 3 Department of Oncology, Yueyang Hospital of Integrated Traditional Chinese and Western Medicine, Shanghai University of Traditional Chinese Medicine, Shanghai, China

**Keywords:** POLR1D, Lung cancer, PI3K, AKT, Cell proliferation

## Abstract

**Aim:**

The most common type of cancer that leads to death worldwide is lung cancer. Despite significant surgery and chemotherapy improvements, lung cancer patient’s survival rate is still poor. The RNA polymerase I subunit D (POLR1D) gene can induce various cancers. A current study reported that POLR1D plays a vital role in cancer prognosis. However, its biological function in the development of lung cancer remains unclear.

**Methods:**

Reverse transcription PCR (RT-PCR) measured the relative POLR1D protein expression level in lung cancer cell lines. Lung cancer cell proliferation, migration, and invasion were analyzed by performing cell counting kit-8 (CCK-8), and transwell. The phosphatidylinositol 3-kinase/serine-threonine kinase (PI3K/AKT) signaling pathway-related protein expressions were examined by Western blotting assay.

**Results:**

POLR1D protein expression was elevated in lung cancer. Lung cancer cell loss-of-function tests showed that POLR1D silencing could attenuate cell viability both in SK-MES-1 and in H2170 cells. Furthermore, silencing POLR1D inhibited SK-MES-1 and H2170 cells proliferation, migration, and invasion. Moreover, SK-MES-1 and H2170 cells’ migration and invasion capacity were potentially suppressed by the knockdown of POLR1D. The progression of multiple cancers has been implicated in the PI3K/AKT pathway. Here, we observed that POLR1D silencing suppressed lung cancer progression by inhibition of the PI3K-Akt pathway.

**Conclusions:**

The study speculated that POLR1D might provide a new potential therapeutic possibility for treating lung cancer patients via targeting PI3K/AKT.

## Introduction

Lung cancer is one of the most severe tumors threatening human health and life worldwide. Its morbidity and mortality have jumped to the forefront of various malignant tumors. The leading causes of its death are the emergence of metastasis and drug resistance, which are the difficulties in the clinical treatment of lung cancer and the hotspot of lung cancer research [1]. Lung cancer is a disease that is diagnosed in over 1.4 million individuals annually [2]. The two primary kinds of lung cancer, small-cell lung carcinoma (SCLC) and non-small-cell lung carcinoma (NSCLC), accounting for 15–20% and 80–85%, respectively [3]. Despite significant advancements in treating lung cancer, the overall survival rate is still below average [4]. As previously stated, early metastases account for a significant amount of the increased mortality [5]. Thus, it is critical to comprehend how Lung cancer manifests and progresses, as more potent treatments are needed indispensably.

A region frequently gained or amplified in Colorectal cancer (CRC) is the long arm of chromosome 13, between 13q and 13q12.2, where the RNA polymerase I subunit D (POLR1D) gene is located. POLR1D encodes a subunit of RNA polymerases I and III, which are crucial for the transcription of small RNA molecules and ribosomal RNA (rRNA) [6, 7]. Treacher-Collins syndrome (TCS), a rare congenital craniofacial developmental abnormality affecting 1 in 50,000 live births, has been linked to the POLR1D mutation [8]. Limitations in ribosome biogenesis brought on by POLR1D loss-of-function affect important cellular processes such as cell division, proliferation, and differentiation [9, 10]. Reduced ribosome biogenesis prevents the proliferation of neuroepithelial cells and results in a reduction of migrating neural crest cells, which contributes to TSC [11]. Accumulating reports suggest that a higher rate of ribosome biogenesis may prognosis the progress of cancer development [12, 13]. POLR1D has been shown to play a critical role in malignancies; however, its mechanism in the etiology of lung cancer has to be further investigated.

The dysregulation of the phosphatidylinositol 3-kinase/serine-threonine kinase (PI3K/AKT) signaling pathway plays a critical role in tumor formation [14]. Cell proliferation signals generated by binding multiple transmembrane receptors and ligands can activate the PI3K/AKT signaling pathway, which is directly related to the proliferation of tumor cells [1]. For instance, by activating the PI3K/AKT pathway, MALAT1 promotes the growth and spread of ovarian cancer [15]. By regulating PI3K/AKT, dysregulated UCA1 can promote the development of gastric cancer [16]. Additionally, through PI3K/AKT, lncRNA AB073614 regulates the growth of colorectal cancer cells [17]. However, the role of POLR1D in the PI3K/AKT signaling pathway remains unknown.

This research explored how POLR1D and PI3K/AKT signaling affect lung cancer development. We herein report that silencing POLR1D can significantly suppress the proliferation and migration of lung cancer cells by inhibiting the PI3K/AKT pathway.

## Materials and methods

### Cell culture

Human embryonic lung fibroblasts (HFL1) and lung cancer cell lines (H2170, H226, SK-MES-1, PC-9, and H1975) were obtained from the American Type Tissue Culture Collection (ATCC; Rockville, MD, USA). The Dulbecco’s modified Eagle’s medium (DMEM, Invitrogen) with 10% fetal bovine serum (FBS) was maintained at 37^0^C in 5% CO_2_. All the cell lines employed in our investigation had been continuously passaged for around 4 months. Mycoplasma PCR Detection Kit (Sigma-Aldrich, St. Louis, MO) was used to determine the mycoplasma contamination.

### Plasmid construct, siRNA, and transfection

The siRNA toward the human POLR1D gene was also purchased from Genepharma with the following sequence: POLR1D-si1: 5’-GAC ACT GTG TGA CAT TTG TA-3’ (targeting 1155–1174 region, NM_001374407.1); POLR1D-si2: 5’-GCC TGA ATG AGC TCA TGA AT-3’ (targeting 1341–1360 region, NM_001374407.1). The siRNA sequence was transfected into SK-MES-1 and H2170 cells using Lipo2000 according to the manufacturer’s instructions. Western blotting was used 48 h after transfection to evaluate transfection effectiveness.

### Cell proliferation assay

Specific immunohistochemically staining methods are used in cell proliferation tests to identify proliferating cells, which are intended to assess the relative rates of cell division within such target tissues. Transfected SK-MES-1 and H2170 cells were plated at a density of 1 × 10^4^ cells/well in 96-well tissue culture plates and cultured for 72 h, and cell growth was assessed by CCK-8 assay (cat. no. E606335-0500, BBI Life Sciences, Shanghai, China).

### Cell migration and invasion assays

Cell migration was evaluated using 24-well Transwell inserts (8-µm pore size, cat. no. 3422, CORNING). A total of 5 × 10^5^ cells in 200µL serum-free DMEM were seeded into the upper chambers of Transwell. The lower chambers were supplied with 500µL DMEM containing 10% FBS. A cotton wool swab was used to delicately remove the cells from the upper surface of the membranes after the membranes had been incubated for 24 h. In contrast, those migrating to the membrane surface’s lower side were fixed and stained with crystal violet (cat. no. E607309, BBI Life Sciences, Shanghai, China). From 10 random fields, the average number of migrating cells was counted (about 200). For the invasion assay, similar procedures were carried out except for the Transwell inserts loaded with Matrigel (cat. no. 354,480, CORNING), and cells were cultured for 48 h.

### Quantitative reverse transcription polymerase chain reaction (RT-qPCR)

TRIzol reagent (Invitrogen) was carried out to extract total RNA. Superscript II reverse transcriptase (Toyobo Life Science, Osaka, Japan) was used to reverse transcription on an RNA template to produce cDNA. SYBR Premix Ex-Taq (Takara Bio, Japan) real-time PCR was performed using an Applied Biosystems 7500 Fast Real-Time PCR System (Applied Biosystems; Foster City, CA, USA). PCR primers included: POLR1D sense (5’‑CTG AAG GCG AGA GGA AGA CAG‑3’) and POLR1D antisense (5’‑GGT ACC TCG AGT CTG AAT GCG‑3’); GAPDH sense (5’‑GGA GCG AGA TCC CTC CAA AAT‑3’) and GAPDH antisense (5’‑GGC TGT TGT CAT ACT TCT CAT GG‑3’). For real-time PCR, the following conditions were used: 40 cycles of 94 °C for 15 s and 58 °C for 30 s each to amplify the sample for 5 min at 94 °C. The relative mRNA levels were determined using the 2-ΔΔCt technique [18].

### Western blotting

Proteins were extracted from ventricular tissue using lysis buffer and measured using a BCA assay kit (Beyotime). SDS-PAGE was applied to separate the total protein (50 µg), which was then transferred to PVDF membranes. The membranes blocked and incubated with primary and secondary antibodies against POLR1D (1:400, ab243591, rabbit polyclonal, Abcam); Cleaved caspase-3 (1:500, ab32042, rabbit monoclonal, Abcam); E-cadherin (1:500, 20874-1-AP, rabbit polyclonal, Proteintech); N-cadherin (1:500, 22018-1-AP, rabbit polyclonal, Proteintech); p-PI3K (1:500, #4228, rabbit polyclonal, Cell Signaling Technology); PI3K (1:500, R22768, rabbit polyclonal, ZenBio); p-AKT (1:500, #4060S, rabbit polyclonal, Cell Signaling Technology); AKT (1:500, #4691, rabbit polyclonal, Cell Signaling Technology) and GAPDH (1:2000, sc-47,724, mouse monoclonal, Santa Cruz) were using the 5% skimmed milk. Then, membranes were washed with a solution of Tris-buffered saline and Tween-20 before being treated with a secondary antibody conjugated to horseradish peroxidase for two hours at room temperature. For repeated use of PVDF membrane, antibody stripping solution (WB6500, NCM Biotech, Suzhou, China) was used. ECL reagents were used to identify protein bands (Amersham Biosciences, UK). ImageJ software was used to examine the protein bands, and GAPDH was employed as an internal control.

### Statistical analysis

The SPSS statistical software (Version 20.0 SPSS Inc.) and GraphPad Prism 6.0 software were used for all statistical analyses. One-way ANOVA was applied to analyze the difference between three or more groups, followed by Student-Newman-Keuls tests. The *p* < 0.05 threshold was used to determine statistical significance.

## Results

### POLR1D protein expression was increased in lung cancer

**Fig. 1:**
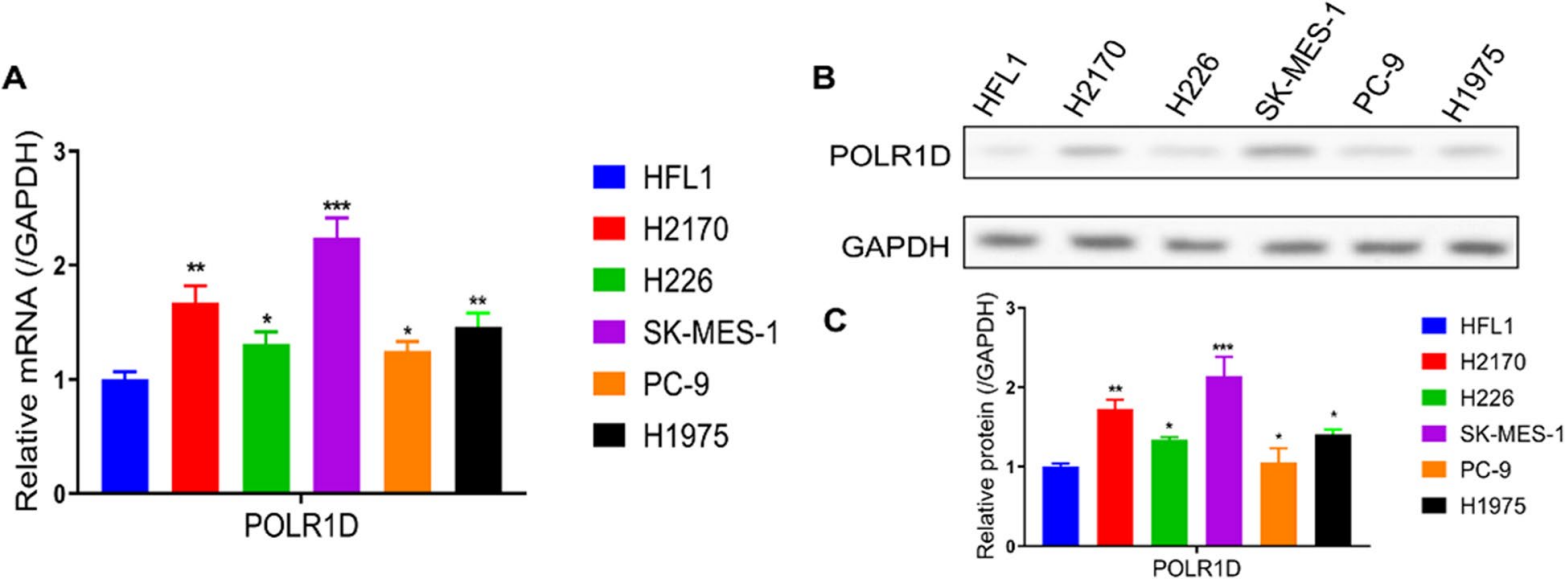
Expression of POLR1D in lung cancer cells. **a** The mRNA expression of POLR1D in human embryonic lung fibroblasts (HFL1) and five-lung cancer cell lines (H2170, SK-MES-1, H226, PC-9, H1975) by RT‑qPCR. **b** The representative bands of POLR1D protein in six-lung cancer cell lines. **c** Quantification of POLR1D protein expression determined by Western blot. Data represent the average of three independent experiments (mean ± SD). *P < 0.05, **P < 0.01, ***P < 0.001 vs. HFL1 group

To investigate the potential role of POLR1D in lung cancer, we examined the expression of POLR1D in human embryonic lung fibroblasts (HFL1) and five lung cancer cell lines (H2170, SK-MES-1, H226, PC-9, H1975) using RT‑qPCR. The results showed that POLR1D expression was markedly increased in lung cancer cells compared to normal human HFL1 cells (Fig. 1a). Furthermore, POLR1D expression in H2170, SK-MES-1, H226, PC-9, H1975, and HFL1 cells was determined via western blot analysis. Consistently, POLR1D was significantly increased in lung cancer cells (Fig. 1b, c). These findings suggested that POLR1D was increased in lung cancer, supporting a potential function in developing lung cancer.

### POLR1D inhibition suppresses lung cancer cells proliferation

**Fig. 2:**
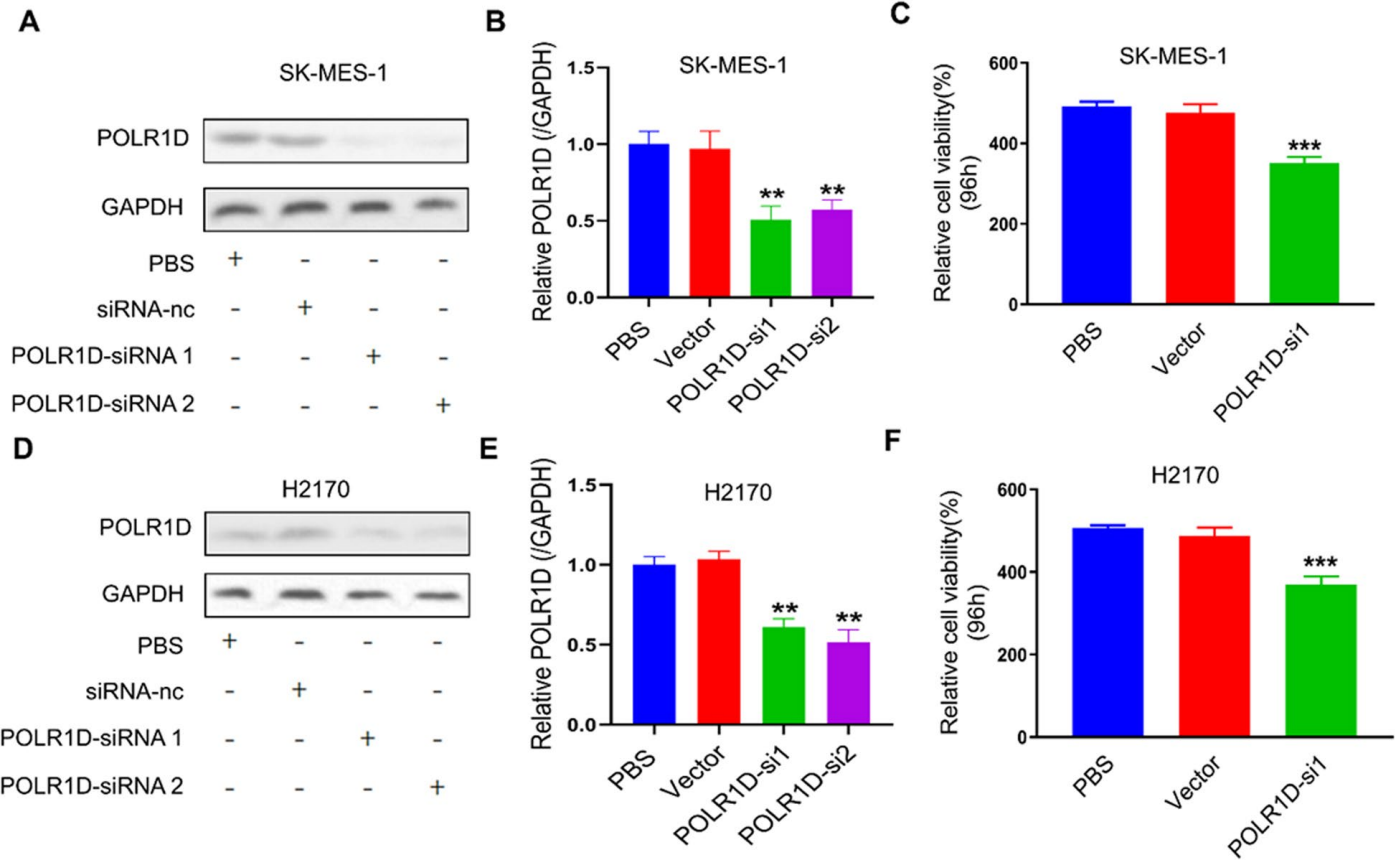
Inhibition of POLR1D in lung cancer cells by siRNA suppresses cell proliferation. **a** Two siRNAs targeting POLR1D mRNA (POLR1D-si1 and POLR1D-si2) were introduced into SK-MES-1 and H2170 cells for transient knockdown of POLR1D. The levels of POLR1D protein were detected by Western blotting in SK-MES-1 and H2170 cells. Expression was normalized against endogenous GAPDH levels. **b**,** c** Quantification of POLR1D protein levels SK-MES-1 and H2170 cells. **d** The levels of POLR1D protein were detected by Western blotting in H2170 cells. **e**,** f** The cell growth rate was suppressed by POLR1D knockdown in SK-MES-1 and H2170 cells detected by CCK-8 assay 72 h after transfection. The information shown is the mean of three separate experiments (mean ± SD). ***P < 0.001 vs. Control group

To transient knockdown of POLR1D, two siRNAs (POLR1D-si1 and POLR1D-si2) targeting POLR1D’s mRNA were introduced into SK-MES-1 and H2170 cells. We observed that the POLR1D protein expression was significantly inhibited in SK-MES-1 and H2170 cells compared with their corresponding controls (Fig. 2a-c). Then, using the CCK-8 assay, we observed that SK-MES-1 and H2170 cells infected with siRNA‐1 and siRNA‐1 of POLR1D potentially decreased cell viability. Moreover, Cell proliferation assay analysis showed that POLR1D knockdown significantly inhibited cell proliferation in the SK-MES-1 and H2170 cell lines (Fig. 2d, e). Thus, the results implied that POLR1D induced lung cancer cell proliferation.

### Downregulation of POLR1D attenuates lung cancer cells migration and invasion

**Fig. 3:**
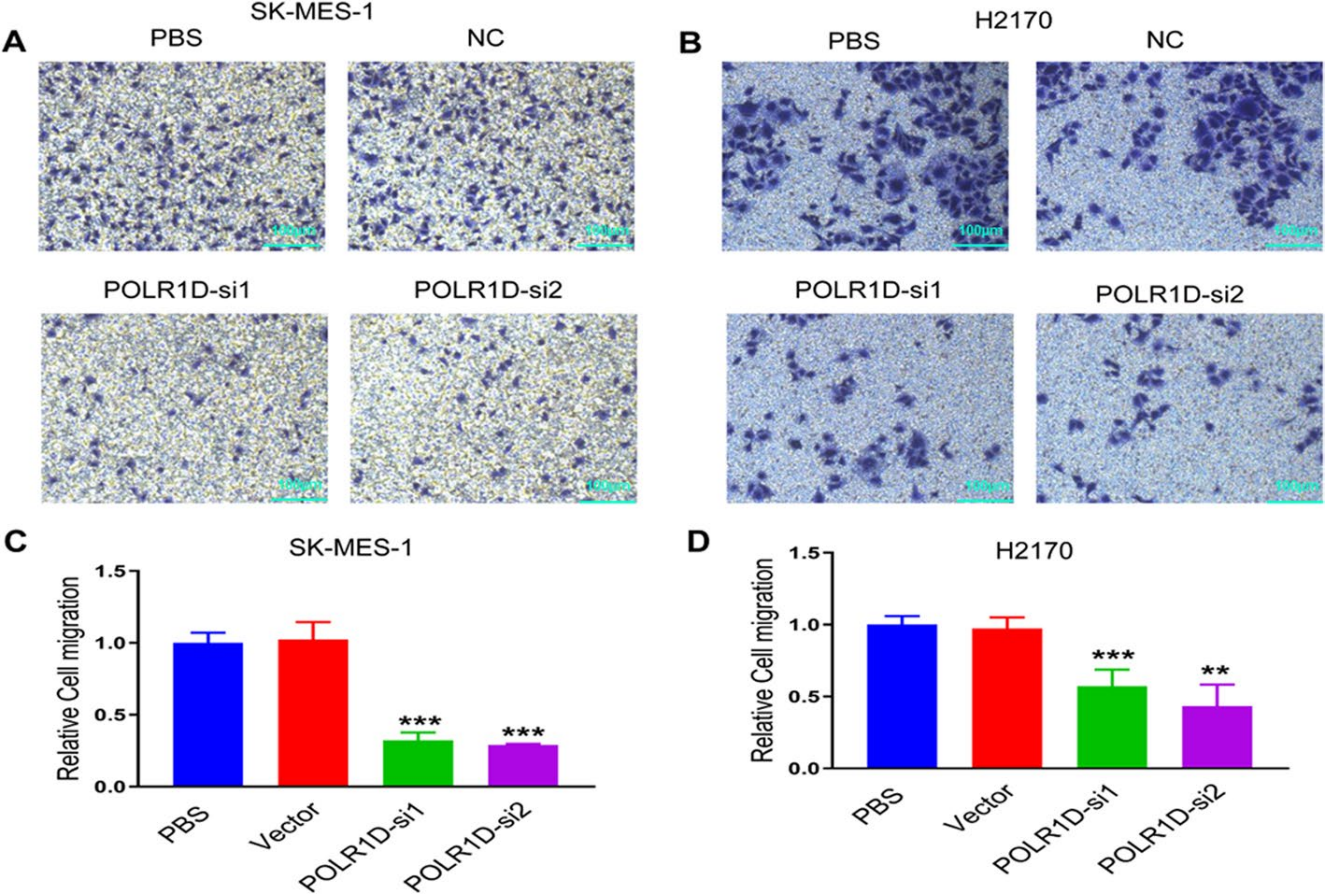
Downregulation of POLR1D inhibits the migration of lung cancer cells. The effects of POLR1D deficiency on cell migration were investigated by Transwell assay in (**a**) SK-MES-1 and (**b**) H2170 cells (scale bar: 50 μm). The relative migrated cells were quantified in (**c**) SK-MES-1 and (**d**) H2170 cells. Data represent the average of three independent experiments (mean ± SD). ***P < 0.001 vs. PBS group

**Fig. 4:**
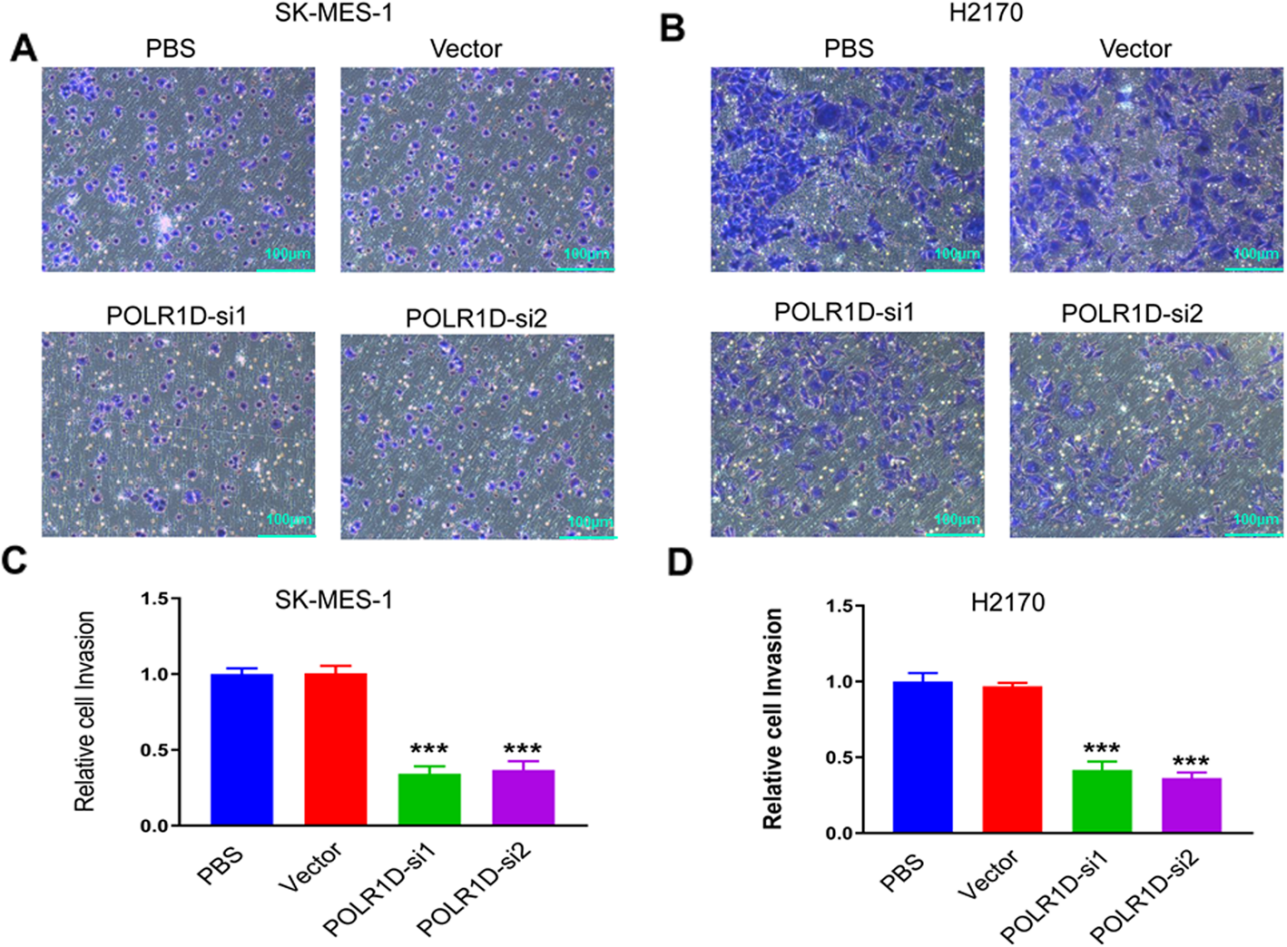
Downregulation of POLR1D inhibits the invasion of lung cancer cells. Knockdown of POLR1D on cell invasion was investigated by Transwell assay in (**a**) SK-MES-1 and (**b**) H2170 cells (scale bar: 50 μm). The relative invaded cells were quantified in (**c**) SK-MES-1 and (**d**) H2170 cells. Data represent the average of three independent experiments (mean ± SD). ***P < 0.001 vs. PBS group

We carried out the Transwell assays to investigate the migration and invasion capability of SK-MES-1 and H2170 cells. The results showed that the downregulation of POLR1D potentially inhibits the migration capability of SK-MES-1 and H2170 lung cancer cells (Fig. 3a, b). The same consistent results were observed after quantification of the relative migrated cells (Fig. 3c, d). Then, a Transwell invasion experiment was performed to see if POLR1D affected lung cancer invasion capacity. The results demonstrated that the knockdown of POLR1D significantly inhibited the invasion ability of SK-MES-1 and H2170 cells (Fig. 4a, b). We then quantified the relative invasion cells and observed the same trends of reduced invasion ability of the SK-MES-1 and H2170 cells (Fig. 4c, d). Thus, the results speculated that POLR1D might suppress lung cancer cell migration and invasion capacity.

### POLR1D silencing suppressed lung cancer progression via inhibition of PI3K-Akt pathway

**Fig. 5:**
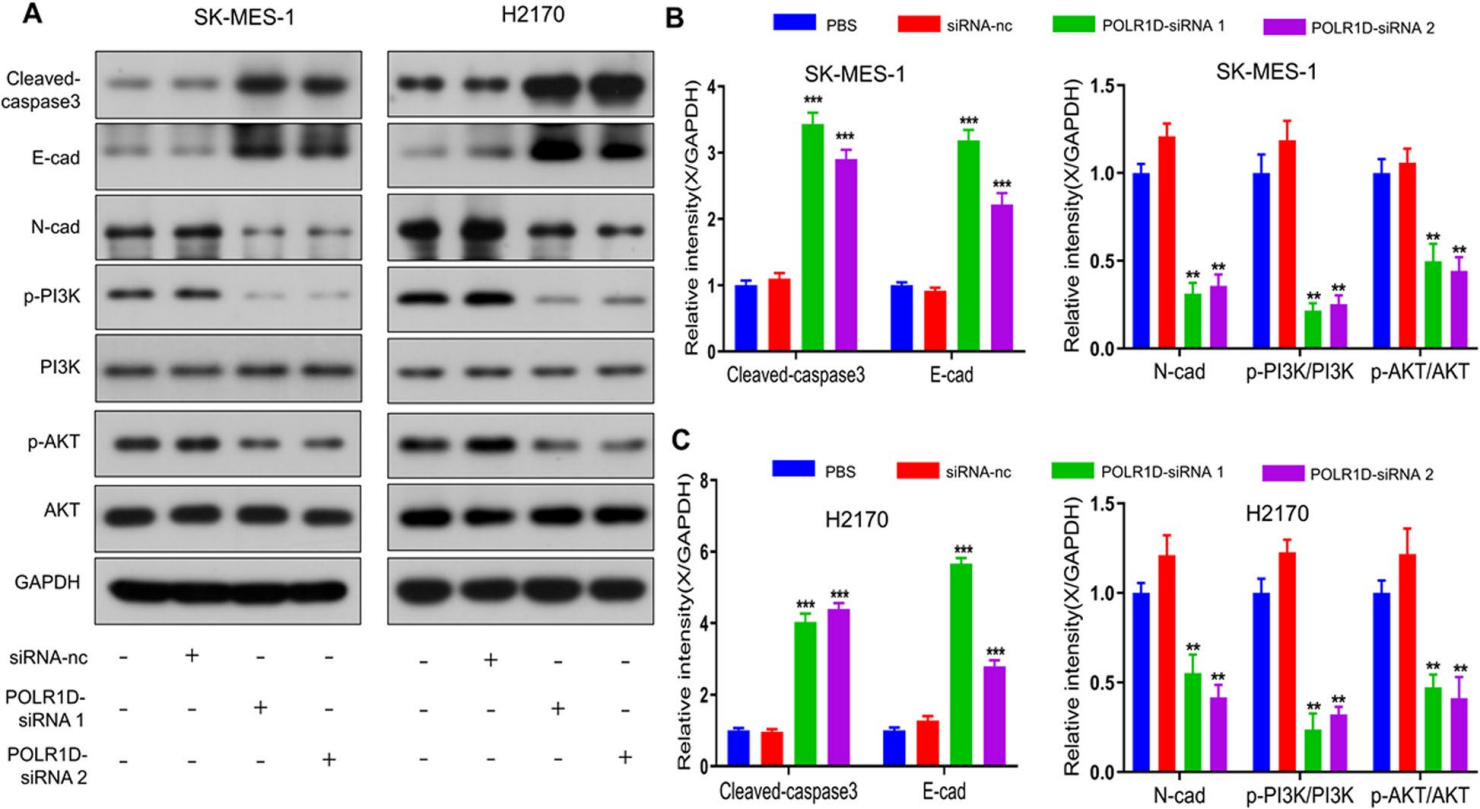
Effects of POLR1D silencing on proteins of EMT and PI3K-AKT pathway. **a** Representative gel blots depicting levels of cleaved caspase-3 (normalized to GAPDH), E-cadherin (normalized to GAPDH), N-cadherin (normalized to GAPDH), phosphorylated PI3K (p-PI3K) and total PI3K (normalized to total PI3K), phosphorylated AKT (p-AKT) and total AKT (normalized to total AKT). These protein blots were quantified in (**b**) SK-MES-1 cells and **c** H2170 cells. Data represent the average of three independent experiments (mean ± SD). **P < 0.01, ***P < 0.001 vs. PBS group

Recent results suggested that the PI3K/AKT pathway is associated with the progression of tumor formation. Western blotting analysis in Fig. 5, revealed that POLR1D silencing potentially reduced the p-AKT expression, whereas it did not affect the expression of total AKT. In addition, POLR1D knockdown in SK-MES-1 and H2170 cells significantly reduced the expression of the p-PI3K protein. Moreover, POLR1D silencing potentially increased the E-cad expression, whereas N-cad expression was reduced both in SK-MES-1 and H2170 cells. These data suggested that silencing of POLR1D impeded lung cancer progression by inhibiting the PI3K/AKT pathway.

## Discussion

Lung cancer patient survival rates are still less, despite improvements in surgical methods [19] and comprehensive treatment [20]. Most of these patients are primarily asymptomatic, and the disease is frequently identified only when it has progressed significantly [21]. There were 610,000 lung cancer fatalities and an estimated 733,000 new cases [22]. Therefore, finding a potential tumor progression marker and an effective therapeutic target in lung cancer is inevitable. Recent increasing evidence suggests that POLR1D is involved in the progression of lung cancer, but understanding its roles remains limited.

The RNA polymerase I and III subunits that POLR1D encoded regulated ribosomal RNA transcription and ribosome biogenesis [23]. Recently reported that the mutation of POLR1D leads to Treacher-Collins syndrome (TCS) [8, 23]. Furthermore, a previous study demonstrated that POLR1D loss-of-function results in ribosome biogenesis disorder, activation of p53-dependent neuroepithelial apoptosis, and reduced neural crest cell (NCC) proliferation. TCS results from this restriction on neural crest cells’ ability to migrate, which is the primary source of the craniofacial skeleton [11]. On the one hand, POLR1D loss-of-function increased the expression of p53, which may have inhibited cell proliferation as a negative regulator of ribosome biogenesis [11, 24]. On the other hand, regular use of the non-steroidal anti-inflammatory drug aspirin may down-regulate ribosome biogenesis and thus lower the risk of colorectal cancer [25]. Since POLR1D is a subunit of RNA polymerase I and III, we had a reasonable hypothesis that it may improve ribosome biogenesis and, as a result, promote cell proliferation in lung cancer (which has higher POLR1D protein expression). To investigate our hypothesis, we explored the role of POLR1D in lung cancer in vitro analysis. POLR1D knockdown in SK-MES-1 and H2170 cells dramatically reduced cell proliferation, migration, and invasion capacity.

The PI3K/AKT pathway plays a critical role in the progression of cancer cells [26]. Oncogenes can activate PI3K, which can accelerate cancer development [27]. AKT, a PI3K downstream effector, involves several cellular processes [28]. Through the PI3K/AKT pathway, lncRNA LINC00152 knockdown inhibits the biological activity of lung cancer [29]. Inhibition of the PI3K/AKT pathway silencing the lncRNA reprogramming regulator can increase lung cancer susceptibility to cisplatin [30]. Additionally, by activating the downstream PI3K/AKT pathway, the long non-coding RNA (lncRNA) highly upregulated in liver cancer (HULC) enhances lung cancer cell development [31]. This study reported that POLR1D could improve lung cancer progression by inhibiting the PI3K/AKT pathway. As we observed, p-AKT protein expression was reduced, whereas there was no change in total AKT expression by POLR1D silencing. p-PI3K protein expression was also markedly decreased by POLR1D knockdown in SK-MES-1 and H2170 cells. Thus, these results suggested that the PI3K/AKT pathway might be associated with POLR1D-regulated lung cancer development.

## Conclusions

In this study, we reported that POLR1D affected the proliferation, migration, and invasion of lung cancer by regulating the PI3K-AKT pathway. High POLR1D protein expression was observed in lung cancer. Accumulating these results demonstrated that POLR1D could be a potential therapeutic target for treating lung cancer. Future research should explore the detailed mechanism by which the POLR1D regulates the PI3K-AKT pathway in lung cancer.

## Data Availability

All data generated or analyzed during this study are included in this article. The datasets generated and/or analysed during the current study are available from the corresponding author on reasonable request.

## References

[1] Zhu B, Zhou X (2011). The study of PI3K/AKT pathway in Lung Cancer Metastasis and Drug Resistance. Chin J Lung Cancer.

[2] Jemal A, Bray F, Center MM, Ferlay J, Ward E, Forman D (2011). Global cancer statistics. Cancer J Clin.

[3] Goldstraw P, Ball D, Jett JR, Le Chevalier T, Lim E, Nicholson AG, Shepherd FA (2011). Non-small‐cell lung cancer. Lancet.

[4] Rosell R, Bivona TG, Karachaliou N (2013). Genetics and biomarkers in personalization of lung cancer treatment. Lancet.

[5] Peters  S, Adjei  AA, Gridelli  C, Reck  M, Kerr  K, Felip  E, ESMO Guidelines Working Group (2012). Metastatic non-small‐cell lung cancer (NSCLC): ESMO Clinical Practice Guidelines for diagnosis, treatmentfollow‐up. Ann Oncol.

[6] de Groen FL, Krijgsman O, Tijssen M, Vriend LE, Ylstra B, Hooijberg E (2014). Gene-dosage dependent overexpression at the 13q amplicon identifies DIS3 as candidate oncogene in colorectal cancer progression. Genes Chromosomes Cancer.

[7] Hermsen M, Postma C, Baak J, Weiss M, Rapallo A, Sciutto A (2002). Colorectal adenoma to carcinoma progression follows multiple pathways of chromosomal instability. Gastroenterology.

[8] Dauwerse JG, Dixon J, Seland S, Ruivenkamp CAL, van Haeringen A, Hoefsloot LH, Peters DJM, Boers AC, Daumer-Haas C, Maiwald R (2010). Mutations in genes encoding subunits of RNA polymerases I and III cause Treacher Collins syndrome. Nat Genet.

[9] Jorgensen P, Rupes I, Sharom JR, Schneper L, Broach JR, Tyers M (2004). A dynamic transcriptional network communicates growth potential to ribosome synthesis and critical cell size. Genes Dev.

[10] Trainor PA, Merrill AE (2014). Ribosome biogenesis in skeletal development and the pathogenesis of skeletal disorders. Biochim Biophys Acta.

[11] Noack WK, Achilleos A, Neben CL, Merrill AE, Trainor PA (2016). The roles of RNA polymerase I and III subunits Polr1c and Polr1d in Craniofacial Development and in zebrafish models of Treacher Collins Syndrome. Plos Genet.

[12] Brighenti E, Calabrese C, Liguori G, Giannone FA, Trere D, Montanaro L, Derenzini M (2014). Interleukin 6 downregulates p53 expression and activity by stimulating ribosome biogenesis: a new pathway connecting inflammation to cancer. Oncogene.

[13] Donati G, Bertoni S, Brighenti E, Vici M, Trere D, Volarevic S, Montanaro L, Derenzini M (2011). The balance between rRNA and ribosomal protein synthesis up- and downregulates the tumour suppressor p53 in mammalian cells. Oncogene.

[14] Pisick E, Jagadeesh S, Salgia R (2004). Receptor tyrosine kinases and inhibitors in lung cancer. Sci World J.

[15] Jin Y, Feng SJ, Qiu S, Shao N, Zheng JH (2017). LncRNA MALAT1 promotes proliferation and metastasis in epithelial ovarian cancer via the PI3K-AKT pathway. Eur Rev Med Pharmacol Sci.

[16] Li C, Liang G, Yang S, Sui J, Yao W, Shen X (2017). Dysregulated lncRNA-UCA1 contributes to the progression of gastric cancer through regulation of the PI3K‐Akt‐mTOR signaling pathway. Oncotarget.

[17] Wang Y, Kuang H, Xue J, Liao L, Yin F, Zhou X (2017). LncRNA AB073614 regulates proliferation and metastasis of colorectal cancer cells via the PI3K/AKT signaling pathway. Biomed Pharmacother.

[18] Livak KJ, Schmittgen TD (2001). Analysis of relative gene expression data using real-time quantitative PCR and the 2 (-Delta Delta C (T)) method. Methods.

[19] Long H, Tan Q, Luo Q, Wang Z, Jiang G, Situ D (2018). Thoracoscopic surgery versus thoracotomy for lung cancer: short-term outcomes of a randomized trial. Ann Thorac Surg.

[20] Bott MJ, Yang SC, Park BJ, Adusumilli PS, Rusch VW, Isbell JM (2019). Initial results of pulmonary resection after neoadjuvant nivolumab in patients with resectable non-small cell lung cancer. J Thorac Cardiovasc Surg.

[21] Nasim F, Sabath BF, Eapen GA (2019). Lung cancer. Med Clin North Am.

[22] Chen W, Zheng R, Baade PD, Zhang S, Zeng H, Bray F (2016). Cancer statistics in China, 2015. CA Cancer J Clin.

[23] Giampietro PF, Armstrong L, Stoddard A, Blank RD, Livingston J, Raggio CL, Rasmussen K, Pickart M, Lorier R, Turner A (2015). Whole exome sequencing identifies a POLRID mutation segregating in a father and two daughters with findings of Klippel-Feil and Treacher Collins syndromes. Am J Med Genet A.

[24] Zhai W, Comai L (2000). Repression of RNA polymerase I transcription by the tumor suppressor p53. Mol Cell Biol.

[25] Camps J, Pitt JJ, Emons G, Hummon AB, Case CM, Grade M, Jones TL, Nguyen QT, Ghadimi BM, Beissbarth T (2013). Genetic amplification of the NOTCH Modulator LNX2 upregulates the WNT/ -Catenin pathway in Colorectal Cancer. Cancer Res.

[26] Dey N, De P, Leyland-Jones B (2017). PI3K‐AKT‐mTOR inhibitors in breast cancers: from tumor cell signaling to clinical trials. Pharmacol Ther.

[27] Yang L, Liu Y, Wang M, Qian Y, Dai X, Zhu Y (2016). Celastrus orbiculatus extract triggers apoptosis and autophagy via PI3K/Akt/mTOR inhibition in human colorectal cancer cells. Oncol Lett.

[28] He F, Chen H, Yang P, Wu Q, Zhang T, Wang C (2016). Gankyrin sustains PI3K/GSK-3beta/beta‐catenin signal activation and promotes colorectal cancer aggressiveness and progression. Oncotarget.

[29] Zhang Y, Xiang C, Wang Y, Duan Y, Liu C, Jin Y, Zhang Y, lncRNA (2017). LINC00152 knockdown had effects to suppress biological activity of lung cancer via EGFR/PI3K/AKT pathway. Biomed Pharmacother.

[30] Shi H, Pu J, Zhou XL, Ning YY, Bai C (2017). Silencing long non-coding RNA ROR improves sensitivity of non‐small‐cell lung cancer to cisplatin resistance by inhibiting PI3K/Akt/mTOR signaling pathway. Tumour Biol.

[31] Liu L, Zhou XY, Zhang JQ, Wang GG, He J, Chen YY (2018). LncRNA HULC promotes non-small cell lung cancer cell proliferation and inhibits the apoptosis by up‐regulating sphingosine kinase 1 (SPHK1) and its downstream PI3K/Akt pathway. Eur Rev Med Pharmacol Sci.

